# Mesoscale Simulations of pH-Responsive Amphiphilic Polymeric Micelles for Oral Drug Delivery

**DOI:** 10.3390/pharmaceutics11120620

**Published:** 2019-11-20

**Authors:** Zhimin Wu, Manzhen Duan, Di Xiong, Can Yang Zhang

**Affiliations:** 1 School of Chemical Engineering, Xiangtan University, Xiangtan 411105, China; 201721581667@smail.xtu.edu.cn (M.D.); xiongdi@xtu.edu.cn (D.X.); 2 Department of Pharmaceutical Sciences, College of Pharmacy and Pharmaceutical Sciences, Washington State University, Spokane, WA 99210, USA

**Keywords:** polymeric micelles, amphiphilic, DPD simulation, self-assembly, structure-property

## Abstract

It is of great significance to study the structure property and self-assembly of amphiphilic block copolymer in order to effectively and efficiently design and prepare drug delivery systems. In this work, dissipative particle dynamics (DPD) simulation method was used to investigate the structure property and self-assembly ability of pH-responsive amphiphilic block copolymer poly(methyl methacrylate-*co*-methacrylic acid)-*b*-poly(aminoethyl methacrylate) (poly(MMA-*co*-MAA)-*b*-PAEMA). The effects of different block ratios (hydrophilic PAEMA segment and pH-sensitive PMAA segment) in copolymer on self-assembly and drug loading capacity including drug distribution were extensively investigated. The increase of hydrophilic PAEMA facilitated the formation of a typical core-shell structure as well as a hydrophobic PMAA segment. Furthermore, the optimal drug-carrier ratio was confirmed by an analysis of the drug distribution during the self-assembly process of block copolymer and model drug Ibuprofen (IBU). In addition, the drug distribution and nanostructure of IBU-loaded polymeric micelles (PMs) self-assembled from precise block copolymer (PMMA-*b*-PMAA-*b*-PAEMA) and block copolymer (poly(MMA-*co*-MAA)-*b*-PAEMA) with random pH-responsive/hydrophobic structure were evaluated, showing that almost all drug molecules were encapsulated into a core for a random copolymer compared to the analogue. The nanostructures of IBU-loaded PMs at different pH values were evaluated. The results displayed that the nanostructure was stable at pH < p*K*_a_ and anomalous at pH > p*K*_a_ which indicated drug release, suggesting that the PMs could be used in oral drug delivery. These findings proved that the amphiphilic block copolymer P(MMA_30_-*co*-MAA_33_)-*b*-PAEMA_38_ with random structure and pH-sensitivity might be a potential drug carrier. Moreover, DPD simulation shows potential to study the structure property of PMs self-assembled from amphiphilic block copolymer.

## 1. Introduction

Various core-shell polymeric nanocarriers self-assembled from amphiphilic block copolymers in aqueous solution have been extensively developed and investigated [1,2,3,4,5,6]. Generally, the inner core can enhance the hydrophobic drug loading efficiency, while the outer hydrophilic shell provides a stable interface between the core and the medium. In the past few decades, pH-sensitive polymeric micelles (PMs) have attracted more and more attention and are widely used as smart drug delivery systems [7,8,9,10,11,12,13,14,15]. For instance, Woitiski et al. introduced positively charged chitosan into the PMs, which significantly increased the residence time of the drug in the intestine and increased the bioavailability [16]. Huang et al. used cationic GSK peptide to modify the chitosan platform, leading to the strong mucoadhesive of drug-loaded systems [17,18]. However, natural chitosan-based copolymers used as oral delivery carriers are limited widely due to the lack of pH sensitivity and mucoadhesivity [19]. Therefore, to address these challenges, the development of multi-functional intelligent nanocarriers has gained vast attention. For instance, acrylic-based copolymers with pH-sensitivity, such as poly(methacrylic acid) (PMAA), are extensively used as oral drug delivery carriers [20,21,22,23]. The polymer PMMA is widely used to fabricate multifunctional nano-scale carriers for drug delivery and controlled release as reported [24,25,26,27]. The hydrophilic cationic polymer poly(2-amino ethyl methacrylate) (PAEMA) has been widely used as a building block for adhesive materials [28]. For example, the block copolymer poly(methyl methacrylate-*co*-methacrylicacid)-*b*-poly(2-amino ethyl methacrylate) poly(MMA-*co*-MAA)-*b*-PAEMA with mucoadhesivity and pH-sensitivity has been successfully synthesized and used as stimuli-responsive carriers for insulin delivery and controlled release [29]. However, it is still a challenge to effectively develop stimuli-responsive PMs for drug delivery and controlled release with desired properties. This is because lots of time, financial and human resources are used in the design, synthesis and characterization of drug delivery systems, such as repeated synthesis of undesired stimuli-responsive copolymer.

Dissipative particle dynamics (DPD), which is a coarse-grained simulation method proposed by Hoogerbrugge and Koelman, can reveal mesoscopic-level information in the formation of PMs [30,31,32,33,34,35,36,37,38]. In the present work, DPD simulation was employed to investigate the self-assembly process, structure-property and stimuli-responsive behavior of multifunctional block copolymers based on copolymer poly(MMA-*co*-MAA)-*b*-PAEMA which was used for oral delivery of insulin in our previous work [29]. Ibuprofen (IBU), a widely used small molecular drug in clinics, was selected as the model drug and encapsulated into the core of PMs via hydrophobic interaction. The self-assembly, structure property and pH-sensitivity of amphiphilic block copolymer with different block ratios and topological structures were thoroughly investigated by DPD simulation. This study explores the structure property of stimuli-responsive block copolymer, and DPD simulation might be an effective method to reasonably design and optimize multifunctional biomaterials for drug delivery and controlled release.

## 2. Materials and Methods

### 2.1. Dissipative Particle Dynamics (DPD) Theory

The DPD simulation method was a computer simulation technique developed by Hoogerbrugge and Koelman [39,40]. This method was based on the fluid dynamics simulation of the soft ball system. It was used to study the dynamic behavior of the fluid by establishing a coarse granulation model in which multiple atoms were replaced by a single bead according to Newton’s equations of motion. DPD introduced long-range hydrodynamic forces directly into its equation of motion, allowing for a more realistic simulation of the dynamics of phase separation and other processes that rely on long-range interactions. DPD is an effective method to simulate complex fluids on mesoscopic time and space scales.

In 1995, Espanol and Warren combined the dissipative particle dynamics method with fluctuation–dissipation theory to derive a new formula for the DPD method [41]. In this model, the interaction between all particles consisted of three parts: conservation, dissipative and random forces, and the weight function of the dissipative and stochastic forces must satisfy the fluctuation–dissipation theorem to make the system satisfy regular statistical rules of ensemble. The conservation force describes the repulsive force between the elastic spheres. The dissipative force describes the viscous resistance between the moving beads. The introduction of the random force was to keep the temperature of the simulation system constant. The introduction of three forces enabled the DPD method to perform simulation calculations well on regular ensembles. Compared to conventional molecular dynamics and Brownian dynamics simulations, it had the advantage that all forces were “soft”, allowing the use of larger time steps and correspondingly shorter simulation times, laying the foundation for DPD simulation methods. Groot and Warren used a stepwise coarse-graining method to correlate the force parameters in the DPD simulation with Flory-Huggins interaction parameters [42], thus, initially solving the interaction forces and specific studies of the simulated systems in the DPD simulation. The core problem of the mutual mapping relationship between system properties greatly promoted the application of DPD methods in complex fluids, especially polymer systems. Pagonabarraga et al. used a macroscopic to mesoscopic refinement method which required assuming the free energy functional expression of the non-uniform system in advance, and then obtained the corresponding conservative force of the coarse granulation system [43]. Trofimov et al. improved and generalized them to multi-component systems [44].

The basic idea of DPD was to replace a part of the molecule or a mass of matter in the fluid with a “bead”, and the free motion velocity and position of the bead conformed to the Newtonian equation of motion [42], as follows,

(1) $$\frac{dr_{i}}{dt}=v_{i},\;m_{i}\frac{dv_{i}}{dt}=f_{i}$$

where, *r_i_*, *v_i_*, *m_i_*, and *f_i_* were the position vector, velocity, mass, and force of bead *i*, respectively. In general, all beads had the same mass *m_i_* (1 DPD unit) [45]. The total subjected force of each particle was equal to its acceleration. This total force consisted of three parts, as follows,

(2) $$f_{i}=\underset{i\neq j}{\sum }\left(F_{ij}^{C}+F_{ij}^{D}+F_{ij}^{R}\right)$$

The forces acting on each bead included the conservative force ($F_{ij}^{C}$), random force ($F_{ij}^{R}$), and dissipative force ($F_{ij}^{D}$), as shown in Equations (3)–(5).

(3) $$F_{ij}^{C}=\left\{\begin{array}{ll} a_{ij}\left(1-r_{ij}\right){\hat{r}}_{ij}, & \left(r_{ij}<r_{c}\right)\\ 0, & \left(r_{ij}\geq r_{c}\right) \end{array}\right.$$

(4) $$F_{ij}^{R}=\frac{\sigma \omega \left(r_{ij}\right){\hat{r}}_{ij}\zeta }{\sqrt{\delta_{t}}}$$

(5) $$F_{ij}^{D}=-\frac{\sigma^{2}{\left(\omega \left(r_{ij}\right)\right)}^{2}}{2kT}\left(\hat{r}\cdot v_{ij}\right){\hat{r}}_{ij}$$

The conservation force for non-bonded particles was defined by soft repulsion. The dissipative force corresponding to a frictional force depended on both the position and relative velocities of the beads. The random force was a random interaction between bead *i* and its neighbor bead *j*. All forces vanished beyond a certain cutoff radius *r*_c_, whose value was usually set to one unit of length in the simulations. The three forces were given by the following formulae:

Where, $r_{ij}=r_{i}-r_{j}$, $r_{ij}=|r_{ij}|$, ${\hat{r}}_{ij}=r_{ij}/\left|r_{ij}\right|$, $v_{ij}=v_{i}-v_{j}$, σ was the noise strength, ζ denoted a randomly fluctuating variable with zero mean and unit variance, and T was the system temperature. The *r*-dependent weight function *ω*(*r*) = (1 − *r*) for *r* < 1 and *ω*(*r*) = 0 for *r* > 1.

The *a_ij_* was the maximum repulsion between bead *i* and bead *j*, depending on the underlying atomistic interaction, and was linearly related to the Flory-Huggins parameters (*χ_ij_*) as shown in Equation (6),

(6) $$a_{ij}=a_{ii}+3.27\chi_{ij}$$

where *a*_i__i_ was equal to 25. The method of calculating the solubility parameter using the molecular dynamics method was used to find the Flory-Huggins parameter [46], as follows,

(7) $$\chi_{ij}=\;\frac{V_{\mathrm{bead}}}{RT}{\left(\delta_{i}-\delta_{j}\right)}^{2}$$

where, *δ_i_* and *δ_j_* were the solubility parameters of the two beads, respectively, and *V*_bead_ was the molar volume of the beads. R was the gas constant whose value was akin to 8.314.

### 2.2. The Coarse-Grained Model and Simulation Parameters

**Figure 1:**
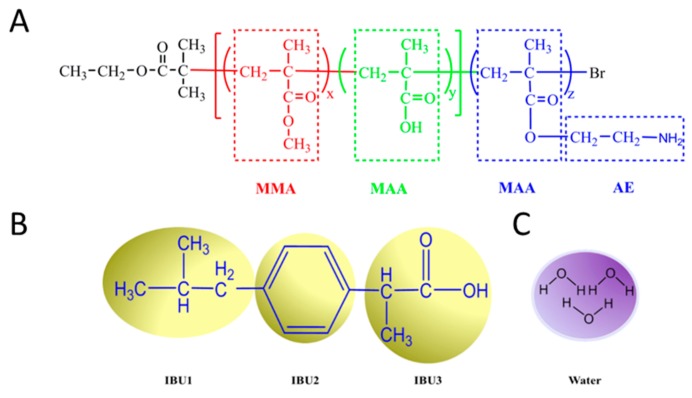
Coarse-grained models of (**A**) block copolymer poly(MMA-*co*-MAA)-*b*-PAEMA, (**B**) ibuprofen, (**C**) water.

Coarse-grained models of block copolymer poly(MMA-*co*-MAA)-*b*-PAEMA, IBU and waters are shown in Figure 1. Each bead was represented by several atoms. The molecular structure of the polymer was divided into three types of beads (MMA, MAA, and AE). PAEMA residue was divided into two types of beads (MAA and AE). The bead of MAA was represented as MAA1 containing carboxylic acid (–COOH) and MAA2 containing carboxylate ions (–COO–). The molecular structure of IBU was also divided into three types of beads (IBU1, IBU2, and IBU3). Three molecules of water were denoted as a bead W.

According to Equations (6) and (7), DPD repulsion parameter a_ij_ was calculated (solubility parameter *δ* was calculated using Amorphous Cell, Discovery, and Blends modules in Materials Studio software with the COMPASS force field). The interaction parameters at 298.15 K used in this simulation are given in Table 1.

A cubic simulation box with periodic boundary condition was applied in all three directions. A box of 40 × 40 × 40 *r*_c_^3^ was used, which was large enough to avoid the finite size effects. In each DPD simulation, simulation steps of 400,000 were adopted to ensure a steady phase. In this work, the coarse-grained beads had an average volume of 95 Å^3^. The bead density used in this work was 3, so the corresponding volume of one bead was 285 Å^3^. Then we could find the physical size of the cut-off radius, *r*_c_ = 6.58 Å. Thus, the box size in our simulation was characterized by effective dimensions of 197.4 × 197.4 × 197.4 Å, which can be used to calculate the length of the simulated structures. All the simulations were performed using DPD program incorporated in the Materials Studio 7.0 software (Accelry Inc.).

## 3. Results

### 3.1. Effect of Hydrophilic and Cationic PAEMA Block

**Figure 2:**
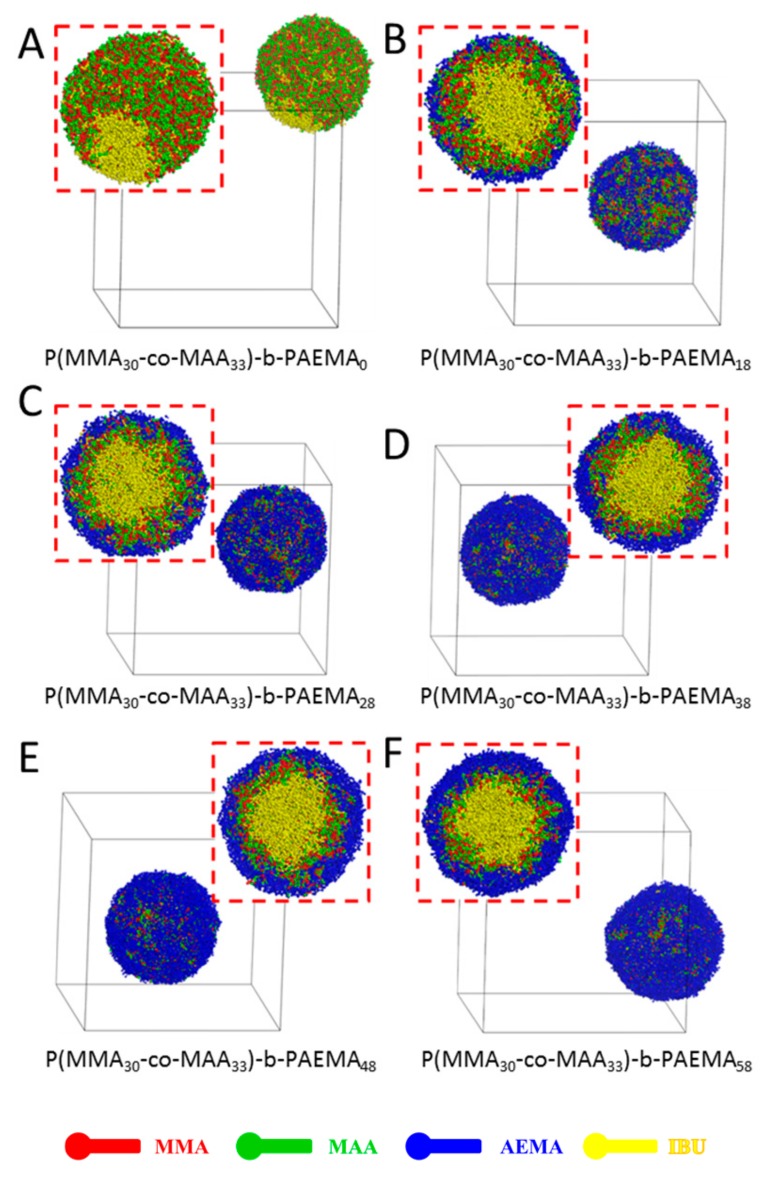
Full-section and cross-section views of IBU-loaded PMs at different ratios of PAEMA block at 400,000 steps during DPD simulation. (poly(MMA_30_-*co*-MAA_33_)-*b*-PAEMA_z_, (**A**) *z* = 0, (**B**) *z* = 18, (**C**) *z* = 28, (**D**) *z* = 38, (**E**) *z* = 48, (**F**) *z* = 58.

We firstly investigated the effect of hydrophilic and cationic PAEMA block on self-assembly of amphiphilic block copolymer poly(MMA-*co*-MAA)-*b*-PAEMA in aqueous solution, as shown in Figure 2. The self-assembly of amphiphilic copolymers was dependent on the amount of the hydrophilic block [47,48,49]. For the block copolymer without PAEMA blocks (poly(MMA_30_-*co*-MAA_33_)-*b*-PAEMA_0_) (Figure 2A), the formed spherical aggregates showed phase separation of the drug from the solid dispersions containing poly(MMA_30_-*co*-MAA_33_) segments, indicating the drugs cannot be loaded in the aggregates. With an increase of the block ratio of the PAEMA segment (Figure 2B–F), PMs coated with hydrophilic and cationic PAEMA block were gradually formed due to the complexation effect between the negatively-charged MAA segment and the positively-charged PAEMA segment. The hydrophobic drug molecules were able to be entrapped in the micellar core as reported [50]. In Figure 2B, although the drugs were encapsulated into the micellar core, the shell was formed by PAEMA, MMA and MAA, suggesting that the IBU-loaded PMs were not stable and cargos might be released rapidly. With an increase of block ratios of the PAEMA segment, the typical onion-like IBU-loaded PMs with IBU core (yellow beads), MMA and MAA middle layer (red and green beads) and PAEMA shell (blue beads) were formed (Figure 2C–F). When the block ratio of the PAEMA segment was 30% (Figure 2C), few MMA and MAA (red and green beads) segments still distributed on the surface, constructing the micellar shell. When this ratio increased to 38% (Figure 2D), the copolymer poly(MMA_30_-*co*-MAA_33_)-*b*-PAEMA_38_ was easily dispersed in water and self-assembled into onion-like IBU-loaded PMs. The IBU molecules were well protected in the core, and the MMA and MAA blocks formed the middle layer. The hydrophilic PAEMA block formed the protective shell. At higher block ratios of PAEMA segment, similar results were found. However, the particle size was increased with the increase of the hydrophilic PAEMA block ratio. Therefore, the copolymer with 38% of the PAEMA segment was used for further study in this work.

### 3.2. Effect of pH-Sensitive PMAA Block

**Figure 3:**
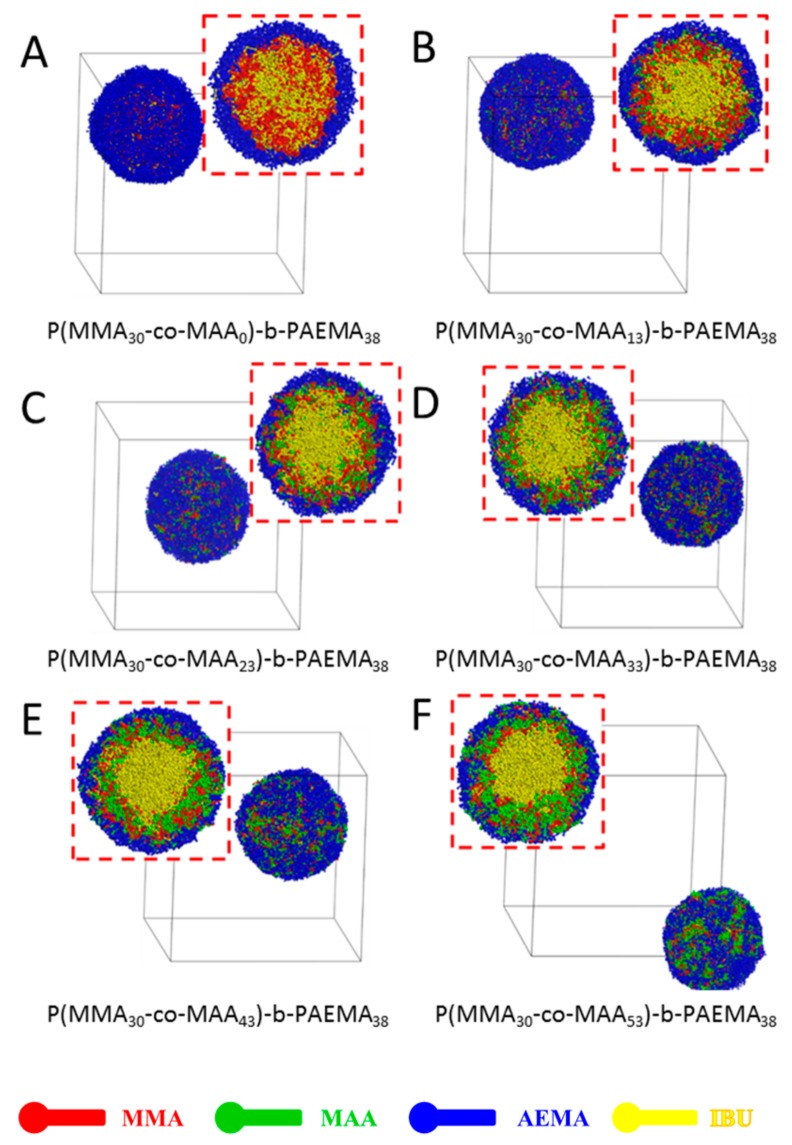
Full-section and cross-section views of IBU-loaded PMs at different ratios of MAA blocks at 400,000 steps during DPD simulation (poly(MMA_30_-*co*-MAA_y_)-*b*-PAEMA_38_, (**A**) *y* = 0, (**B**) *y* = 13, (**C**) *y* = 23, (**D**) *y* = 33, (**E**) *y* = 43, (**F**) *y* = 53.

Next, the effect of pH-sensitive PMAA block on the self-assembly of IBU-loaded PMs was studied, as shown in Figure 3. The morphological appearance of poly(MMA-*co*-MAA)-*b*-PAEMA PMs with MAA numbers of 0, 13, 23, 33, 43, and 53 (i.e., MAA fraction of 0, 18%, 25%, 33%, 38%, and 44%) were visualized using DPD method. It could be observed that all types of PMs presented a spherical morphology with a typical core-shell structure. As seen in Figure 3A, IBU-loaded PMs self-assembled from poly(MMA_30_-*co*-MAA_0_)-*b*-PAEMA_38_ showed a core-shell structure. The micellar shell was formed by PAEMA segment, and the micellar core was composed of an MMA segment (red beads) and IBU molecules (yellow beads). Furthermore, the MMA segments and IBU molecules were distributed randomly in the core. With an increase of the block ratio of the MAA segment, the middle layer was gradually formed by MMA and MAA segments (red and green beads) (Figure 3B–F). In Figure 3B,C, few MMA segments were distributed in the core of IBU-loaded PMs, resulting in reduced drug loading content. Moreover, some IBU molecules were distributed in the middle layer. When the block ratio of the MAA segment was 33% (Figure 3D), the typical onion-like IBU-loaded PMs were formed, indicating reasonable drug loading content and pH-triggered drug release profile. When this ratio was higher than 33% (Figure 3E,F), few MMA and MAA segments (red and green beads) were found in the shell of IBU-loaded PMs, leading to burst drug release. Therefore, IBU-loaded PMs self-assembled from the block copolymer with 33% of the MAA segment would be optimal for formulation.

### 3.3. Effect of Mole Fraction of Drug in Feed

**Figure 4:**
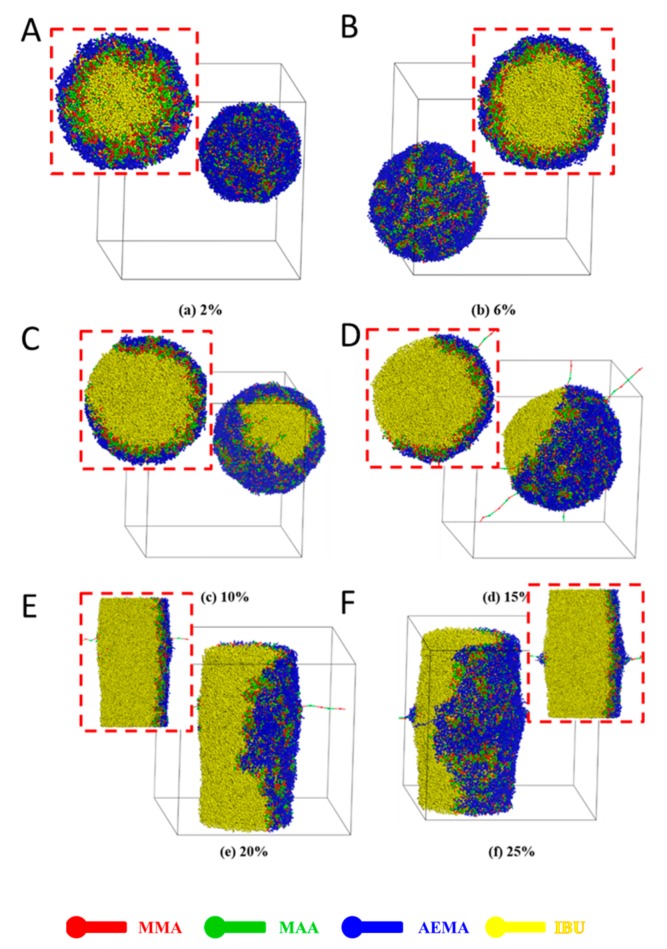
Full-section and cross-section views of IBU-loaded system self-assembled from amphiphilic block copolymer poly(MMA_30_-*co*-MAA_33_)-*b*-PAEMA_38_ together with different mole fraction of IBU in aqueous solution (**A**) 2%, (**B**) 6%, (**C**) 10%, (**D**) 15%, (**E**) 20%, (**F**) 25%.

During the self-assembly process of system, there were another two elements: water and drug. Here, the effect of fraction of water on the self-assembly of the system was firstly studied, as shown in Figure S1. It has been reported that the shape of the amphiphilic copolymer NPs depended on the water content [51]. As shown in Figure S1, the effect of the mole fraction of water in solutions on the formation of IBU-loaded PMs was investigated. When the molar fraction of water was 60% (Figure S1A), the system formed a sandwich-like structure, and the drugs (yellow beads), MMA (red beads) segment and MAA (green beads) segment self-assembled into the middle layer. When the mole fraction of water was increased to 67% (Figure S1B), the sandwich-like structure began to rupture. When the mole fraction of water was further increased (Figure S1C,D), a columnar structure was gradually formed. When the water content was increased above 80% (Figure S1E–I), the copolymer was completely phase separated from water to form spherical PMs. The simulation results showed that the mole fraction of water in the solution influenced a balance between the water and block copolymer, resulting in different structures. Next, the effect of the fraction of the drug in the feed on self-assembly of the system was investigated. The self-assembly of the drug-loaded system with different drug contents (2 to 25%, mole/mole) was simulated by the DPD method, as shown in Figure 4. At 2% IBU in the feed, the drug molecules were able to be well encapsulated into the micellar core, but few MMA (red beads) and MAA (green beads) segments were found in the core (Figure 4A) which indicated more IBU molecules could be loaded, resulting in low drug-loading capacity. When the ratio of the drug was 6%, the typical three-layered onion-like IBU-loaded PMs could be observed (Figure 4B). Almost all IBU molecules were well encapsulated in the micellar core. Clear MMA and MAA segments-constructed middle layers and PAEMA segment-constructed shells were observed. When the ratio of drug in the feed was higher than 6% (Figure 4C–F), the drug molecules undergoing the phase separation were not encapsulated in the core. Furthermore, the spherical shape was changed to irregular column shape. Therefore, 6% IBU in the feed was the optimal formulation during preparation of IBU-loaded PMs.

### 3.4. Self-Assembly Ability

Amphiphilic block copolymers were widely used to prepare nano-size carriers (e.g., polymeric micelles) via self-assembly. Here, the self-assembly process of block copolymer poly(MMA_30_-*co*-MAA_33_)-*b*-PAEMA_38_ (used as oral delivery of insulin in our previous work [29]) in aqueous solution was firstly investigated by DPD simulation, as shown in Figure S2A. At the beginning of the self-assembly (10 steps), many small clusters with different particle sizes were randomly distributed in water. With the clustering process (1000–10,000 steps), the broad size distribution of particles indicated the formation of large aggregates produced from the clustering of the poly(MMA-*co*-MAA)-*b*-PAEMA blocks via the hydrogen bonds. Similarly, several large spherical aggregates were also observed at the simulation step from 30,000 to 150,000. At 200,000 steps, individual PM with spherical morphology was found. With extra simulation steps (300,000 and 400,000 steps), the PM was stabilized and the morphology was not changed significantly. Furthermore, the cross-section view of PM at 400,000 steps is shown in Figure S2B. It is observed that the PM exhibited a typical core-shell architecture with the poly(MMA-*co*-MAA) core (red and green beads) and surrounded the PAEMA shell (blue beads). These findings indicated that the poly(MMA_30_-*co*-MAA_33_)-*b*-PAEMA_38_ was able to self-assemble into PM by coalescence of small clusters in aqueous solution. It was consistent with previous studies of direct imaging of micelle formation that micelles grew mainly via a gradual attachment of copolymer molecules [52].

**Figure 5:**
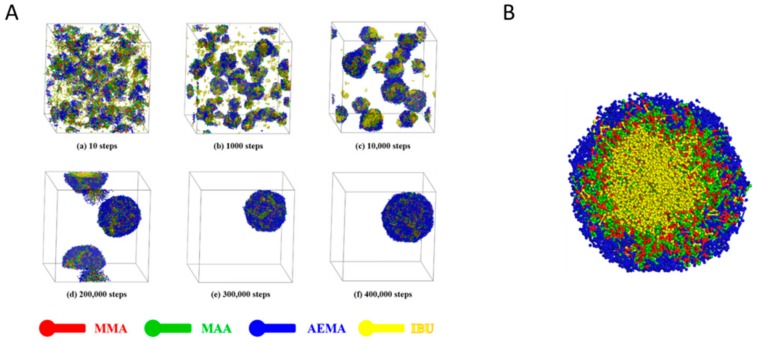
(**A**) Self-assembly process of IBU-loaded poly(MMA_30_-*co*-MAA_33_)-*b*-PAEMA_38_ PMs with 6% IBU in aqueous solution. (**B**) Cross-section view of IBU-loaded poly(MMA_30_-*co*-MAA_33_)-*b*-PAEMA_38_ PMs with 6% IBU at 400,000 steps during DPD simulation.

Next, the self-assembly of block copolymer and model drug IBU was studied by DPD simulation, as shown in Figure 5A. It was clear that the self-assembly process of IBU-loaded PMs was similar to those of the blank PMs (Figure S2A). At 300,000 steps, typical and individual IBU-loaded PM were formed, and compact and stable IBU-loaded PM were received at 400,000 steps. The results indicated that the small-molecule hydrophobic drugs did not influence the self-assembly ability of amphiphilic copolymer in aqueous medium, and could co-self-assemble into PM together with the copolymer molecules. The amphiphilic copolymers cluster could readily absorb onto hydrophobic surfaces, which increased the solubility of insoluble drugs. Figure 5B showed the cross-section view of IBU-loaded PM at 400,000 steps. It was observed that the IBU molecules (yellow beads) could be loaded into the micellar core during the process of self-assembly. In addition, the significant volume expansion (nearly two-fold) was observed after loading of IBU, suggesting high drug-loading ability due to the relatively loose structure of the micellar core based on poly(MMA-*co*-MAA) block. The random coil of the poly(MMA-*co*-MAA) block had more freely exposed carboxyl groups that were able to facilitate H-bonding interactions between hydrophobic chains or hydrophobic chains and drug molecules, which would cause additional entrapment of IBU molecules and provide extra micelle stability simply through a physical interaction.

### 3.5. Effect of Topological Structure of Copolymer

**Figure 6:**
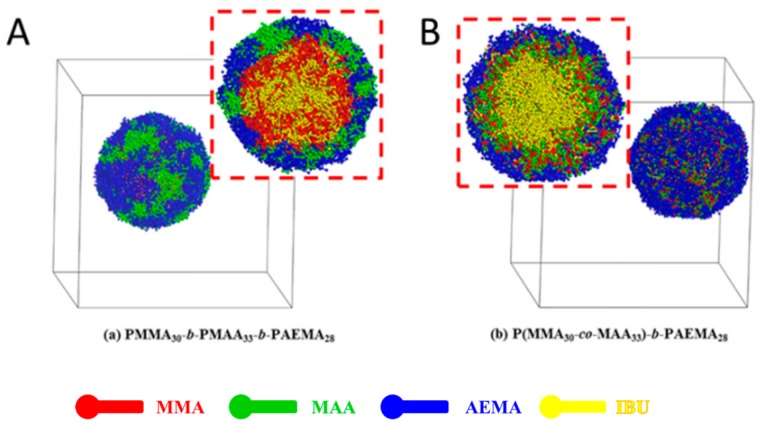
Full-section and cross-section views of IBU-loaded PMs self-assembled from precise line block copolymer (**A**) or random block copolymer (**B**) together with IBU.

Next, the effect of the topological structure of the copolymer on self-assembly and drug distribution of IBU-loaded PMs was studied. Random block copolymer (poly(MMA-*co*-MAA)-*b*-PAEMA) and precise linear block copolymer (PMMA-*b*-PMAA-*b*-PAEMA) (chemical structures were showed in Figure S3) were used to complete the DPD simulation, and the results are shown in Figure 6. As reported, when the thermodynamic properties of two or more blocks in the core were incompatible, the micellar core was segregated into different phases, resulting in “spheres in spheres” (core-shell model, onion-like) and “spheres on spheres” (raspberry-like) [53,54,55]. Therefore, IBU-loaded PMs self-assembled from the precise linear block copolymer, which showed raspberry-like morphology due to MMA and MAA blocks in sequence (Figure 6A), while PMs based on random block copolymers exhibited onion-like PMs (Figure 6B). In Figure 6A, the micellar core was formed by IBU molecules (yellow beads) and MMA (red beads). By contrast, the micellar core of onion-like PMs was formed by IBU molecules. PMMA and PMAA self-assembled into the middle layer, resulting in better protection of encapsulated drugs and pH-triggered drug release profile. In addition, the onion-like PMs with PAEMA shell (blue beads) showed a much higher positive charge in comparison to raspberry-like ones with a hybrid shell (blue and green beads), leading to higher mucoadhesivity. The results suggested that the random topological structure of the block copolymer possessed higher potential for oral drug delivery compared with precise line analogue.

### 3.6. Effect of pH in Solution

**Figure 7:**
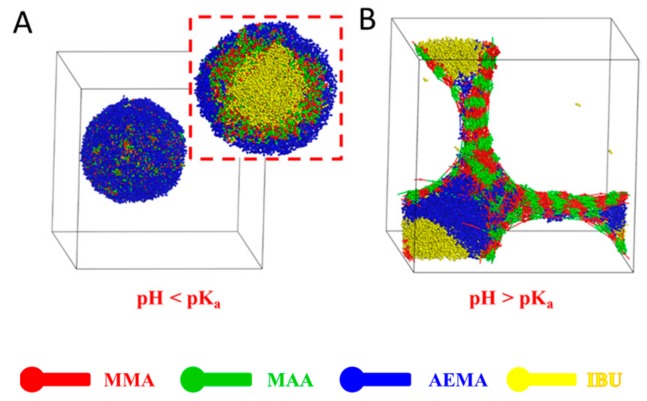
Morphologies of IBU-loaded system self-assembled from amphiphilic block copolymer poly(MMA_30_-*co*-MAA_33_)-*b*-PAEMA_28_ together with IBU (6%) after incubation at different pH values (**A**) pH < p*K*_a_, (**B**) pH > p*K*_a_.

The aggregate morphologies of IBU-loaded PMs at different pH values were further studied by DPD simulation, as shown in Figure 7. When the pH was lower than the p*K*_a_ of the pH-sensitive PMAA segment, copolymer molecules were able to self-assemble into a typical core-shell structure together with IBU molecules with spherical morphology, resulting in three-layered onion-like PMs (Figure 7A). As seen in Figure 7A (cross-section view), IBU molecules (yellow beads) were encapsulated into the micellar core, and PMMA (red beads) and PMAA (green beads) segments formed the middle layer. The hydrophilic PAEMA (blue beads) segments self-assembled into a micellar shell. When the pH was higher than the p*K*_a_ of the pH-sensitive PMAA segment (Figure 7B), the carboxylic acid residues in the PMAA segments lost protons and were ionized, leading to transformation of the solubility from hydrophobicity to hydrophilicity that broke the hydrophilicity–hydrophobicity balance. Therefore, the dismicellization of IBU-loaded PMs was observed. The curled poly(MMA-*co*-MAA) chain stretched and the drug was released from the system. Due to the fact that the p*K*_a_ of the carboxyl group in the PMAA segment was about pH 5–6, the PMAA block was dissolved in the simulated intestinal fluid and was stable under acidic conditions of simulated gastric fluid. In summary, the IBU-loaded PMs were stable, and drug molecules were well protected in the micellar core at pH < p*K*_a_. However, the typical core-shell structure was destroyed, showing an irregular nanostructure at pH > p*K*_a_. These findings indicated that the encapsulated cargos were stable in acidic conditions of gastric juice and released triggered by basic pH value in intestinal tract, suggesting that the PMs could be used in oral drug delivery.

## 4. Discussions and Conclusions

In this paper, the structure property and self-assembly of amphiphilic block copolymer with pH sensitivity were thoroughly investigated by DPD simulation. The effects of different block ratios on structure property (drug loading capacity and drug distribution) and self-assembly were studied. The block copolymer with 38% hydrophilic PAEMA block and 33% pH-sensitive PMAA block (poly(MMA_30_-*co*-MAA_33_)-*b*-PAEMA_38_) showed the most reasonable self-assemble ability, resulting in stable and compact PMs with desired pH-sensitivity. Next, 6% IBU in feed was confirmed as the optimal formulation to prepare IBU-loaded PMs by analysis of drug loading capacity and distribution as well as morphology of systems. The structure property of the topological structure of the copolymer was also studied by DPD simulation. The findings show that the copolymer with a random structure (poly(MMA-*co*-MAA)-*b*-PAEMA) can self-assemble into IBU-loaded PMs together with IBU molecules, showing an onion-like structure with an IBU-formed micellar core, PMMA and PMAA segments-formed middle layer, and PAEMA segment-formed shell. In addition, the optimal preparation method of IBU-loaded PMs in aqueous solutions (the ratios of water and copolymer) was also confirmed. The pH sensitivity of IBU-loaded PMs was investigated by the analysis of the structure of the system at different pH values. The results show that the drug molecules can be explored outside at basic condition (pH > p*K*_a_), indicating the encapsulated drugs could be released triggered by pH in intestinal tract. In summary, the designed amphiphilic copolymer with pH sensitivity in this work might be employed as a potential oral drug delivery nanocarrier. What is more, the DPD simulation could be a useful method to explore the structure property of amphiphilic block copolymer, including self-assembly, drug loading capacity and distribution based on different block ratios and topological structures.

## Figures and Tables

**Table 1:** Repulsive Parameters Used in DPD Simulations (unit: kT).

*a*_ij_	W	IBU1	IBU2	IBU3	MMA	MAA	AE	MAA^−1^
W	25							
IBU1	144.17	25						
IBU2	97.50	30.85	25					
IBU3	106.80	29.03	25.06	25				
MMA	116.88	28.97	25.12	25.01	25			
MAA	71.41	43.82	30.13	30.48	31.40	25		
AE	90.67	38.11	25.02	30.75	25.44	27.65	25	
MAA^−1^	98.92	482.09	372.07	412.66	436.81	308.32	342.01	25

## Supplementary Materials

The following are available online at https://www.mdpi.com/1999-4923/11/12/620/s1, Figure S1: Morphologies of IBU-loaded polymeric NPs self-assembled from copolymer P(MMA30-co-MAA33)-b-PAEMA38 in aqueous solution with different mole fraction of water: (**A**) 60%; (**B**) 67%; (**C**) 70%; (**D**) 75%; (**E**) 80%; (**F**) 89%; (**G**) 90%); (**H**) 91%; (**I**) 97%, Figure S2: (**A**) Self-assembly process of copolymer P(MMA_30_-*co*-MAA_33_)-*b*-PAEMA_38_ PMs in aqueous solution (**B**) Cross-section view of P(MMA_30_-*co*-MAA_33_)-*b*-PAEMA_38_ PMs at 400,000 steps, Figure S3: Chemical structures of random block copolymer P(MMA-*co*-MAA)-*b*-PAEMA (**A**) and precise line block copolymer PMMA-*b*-MAA-*b*-PAEMA (**B**).

## References

[1] Ye T., Shirui M. (2012). Amphiphilic polymeric micelles as the nanocarrier for peroral delivery of poorly soluble anticancer drugs. Expert Opin. Drug Deliv..

[2] Raveendran R., Sharma C.P. (2018). Chapter 12—Polymeric micelles: Smart nanocarriers for anticancer drug delivery. Drug Delivery Nanosystems for Biomedical Applications.

[3] Chen W., Zhou S., Ge L., Wu W., Jiang X. (2018). Translatable high drug loading drug delivery systems based on biocompatible polymer nanocarriers. Biomacromolecules.

[4] Pandey B., Patil N.G., Bhosle G.S., Ambade A.V., Gupta S.S. (2019). Amphiphilic glycopolypeptide star copolymer-based cross-linked nanocarriers for targeted and dual-stimuli-responsive drug delivery. Bioconjug. Chem..

[5] Wang D., Wang J., Huang H., Zhao Z., Gunatillake P.A., Hao X. (2019). Brush-shaped RAFT polymer micelles as nanocarriers for a ruthenium (II) complex photodynamic anticancer drug. Eur. Polym. J..

[6] Xu M., Zhang C.Y., Wu J., Zhou H., Bai R., Shen Z., Deng F., Liu Y., Liu J. (2019). PEG-detachable polymeric micelles self-assembled from amphiphilic copolymers for tumor-acidity-triggered drug delivery and controlled release. ACS Appl. Mater. Interfaces.

[7] Liu H., Chen H., Cao F., Peng D., Chen W., Zhang C. (2019). Amphiphilic block copolymer poly(acrylic acid)-*b*-polycaprolactone as a novel pH-sensitive nanocarrier for anti-cancer drugs delivery: In-Vitro and in-vivo evaluation. Polymers.

[8] Mahmoodzadeh F., Hosseinzadeh M., Jannat B., Ghorbani M. (2019). Fabrication and characterization of gold nanospheres-cored pH-sensitive thiol-ended triblock copolymer: A smart drug delivery system for cancer therapy. Polym. Adv. Technol..

[9] Mattu C., Brachi G., Ciardelli G., Ciofani G. (2018). Smart polymeric nanoparticles. Smart Nanoparticles for Biomedicine.

[10] Wang Z., Deng X., Ding J., Zhou W., Zheng X., Tang G. (2018). Mechanisms of drug release in pH-sensitive micelles for tumour targeted drug delivery system: A review. Int. J. Pharm..

[11] Yang Y., Wang Z., Peng Y., Ding J., Zhou W. (2019). A smart pH-sensitive delivery system for enhanced anticancer efficacy via paclitaxel endosomal escape. Front. Pharmacol..

[12] Li Y., Bui Q.N., Duy L.T.M., Yang H.Y., Lee D.S. (2018). One-Step preparation of pH-responsive polymeric nanogels as intelligent drug delivery systems for tumor therapy. Biomacromolecules.

[13] Jafarzadeh-Holagh S., Hashemi-Najafabadi S., Shaki H., Vasheghani-Farahani E. (2018). Self-assembled and pH-sensitive mixed micelles as an intracellular doxorubicin delivery system. J. Colloid Interface Sci..

[14] Qu J., Peng S., Wang R., Yang S.T., Zhou Q.H., Lin J. (2019). Stepwise pH-sensitive and biodegradable polypeptide hybrid micelles for enhanced cellular internalization and efficient nuclear drug delivery. Colloid Surf. B.

[15] Nguyen V.P., Phi P.Q., Choi S.T. (2019). Tribological Behavior of Grafted Nanoparticle on Polymer-Brushed Walls: A Dissipative Particle Dynamics Study. ACS Appl. Mater. Inter..

[16] Woitiskia C.B., Neufeld R.J., Veiga F., Carvalho R.A., Figueiredo I.V. (2010). Pharmacological effect of orally delivered insulin facilitated by multilayered stable nanoparticles. Eur. J. Pharm. Sci..

[17] Jin Y., Song Y., Zhu X., Zhou D., Chen C., Zhang Z., Huang Y. (2012). Goblet cell-targeting nanoparticles for oral insulin delivery and the influence of mucus on insulin transport. Biomaterials.

[18] Lin Y.H., Sonaje K., Lin K.M., Juang J.H., Mi F.L., Yang H.W., Sung H.W. (2008). Multi-ion-crosslinked nanoparticles with pH-responsive characteristics for oral delivery of protein drugs. J. Control. Release.

[19] Philibert T., Lee B.H., Fabien N. (2017). Current status and new perspectives on chitin and chitosan as functional biopolymers. Appl. Biochem. Biotech..

[20] Luo Y.L., Xu F., Feng Q.S., Chen Y.S., Ma C. (2010). Preparation and characterization of PMAA/MWCNTs nanohybrid hydrogels with improved mechanical properties. J. Biomed. Mater. Res. B Appl. Biomater..

[21] Maroni A., Moutaharrik S., Zema L., Gazzaniga A. (2017). Enteric coatings for colonic drug delivery: State of the art. Expert Opin. Drug Deliv..

[22] Liu L., Zeng J., Zhao X., Tian K., Liu P. (2017). Independent temperature and pH dual-responsive PMAA/PNIPAM microgels as drug delivery system: Effect of swelling behavior of the core and shell materials in fabrication process. Colloid Surf. A.

[23] Liu L., Yao W.D., Rao Y.F., Lu X.Y., Gao J.Q. (2017). pH-Responsive carriers for oral drug delivery: Challenges and opportunities of current platforms. Drug Deliv..

[24] Zhong J.X., Clegg J.R., Ander E.W., Peppas N.A. (2018). Tunable poly(methacrylic acid)-*co*-acrylamide) nanoparticles through inverse emulsion polymerization. J. Biomed. Mater. Res. A.

[25] Simões M.C.R., Cragg S.M., Barbu E., De Sousa F.B. (2019). The potential of electrospun poly(methyl methacrylate)/polycaprolactone core–sheath fibers for drug delivery applications. J. Mater. Sci..

[26] Sahu A., Solanki P., Mitra S. (2018). Curcuminoid-loaded poly(methyl methacrylate) nanoparticles for cancer therapy. Int. J. Nanomed..

[27] Yang Z., Zou H., Liu H., Xu W., Zhang L. (2019). Self-assembly and drug release control of dual-responsive copolymers based on oligo(ethylene glycol)methyl ether methacrylate and spiropyran. Iran. Polym. J..

[28] Bisht G., Zaidi M.G.H., Biplab K.C. (2018). In vivo acute cytotoxicity study of poly(2-amino ethyl methacrylate-*co*-methylene bis-acrylamide) magnetic composite synthesized in supercritical CO_2_. Macromol. Res..

[29] Hu W.Y., Wu Z.M., Yang Q.Q., Liu Y.J., Li J., Zhang C.Y. (2019). Smart pH-responsive polymeric micelles for programmed oral delivery of insulin. Colloid. Surface. B.

[30] Zhao T., Wang X. (2014). Distortion and flow of nematics simulated by dissipative particle dynamics. J. Chem. Phys..

[31] Patterson K., Lisal M., Colina C.M. (2011). Adsorption behavior of model proteins on surfaces. Fluid Phase Equilibr..

[32] Xu J., Zhang J., Xiong D., Lin W., Wen L., Zhang L. (2019). Enhanced stability of crosslinked and charged unimolecular micelles from multigeometry triblock copolymers with short hydrophilic segments: Dissipative particle dynamics simulation. Soft Matter.

[33] Ramezani M., Shamsara J. (2016). Application of DPD in the design of polymeric nano-micelles as drug carriers. J. Mol. Graph. Model..

[34] Wu W.S., Yi P., Zhang J., Cheng Y., Li Z., Hao X., Chen Q. (2019). 4/6-Herto-arm and 4/6-mikto-arm star-shaped block polymeric drug-loaded micelles and their pH-responsive controlled release properties: A dissipative particle dynamics simulation. Phys. Chem. Chem. Phys..

[35] Gao J., Wang P., Wang Z., Li C., Sun S., Hu S. (2019). Self-assembly of DCPD-loaded cross-linked micelle from triblock copolymers and its pH-responsive behavior: A dissipative particle dynamics study. Chem. Eng. Sci..

[36] Anderson R.L., Bray D.J., Del Regno A., Seaton M.A., Ferrante A.S., Warren P.B. (2018). Micelle formation in alkyl sulfate surfactants using dissipative particle dynamics. J. Chem. Theory Comput..

[37] Lin W., Yang C., Xue Z., Huang Y., Luo H., Zu X., Zhang L., Yi G. (2018). Controlled construction of gold nanoparticles in situ from β-cyclodextrin based unimolecular micelles for in vitro computed tomography imaging. J. Colloid Interface Sci..

[38] Xu J., Wang Z.K., Gao J., Li C., Sun S., Hu S. (2017). Dissipative particle dynamics simulations reveal the pH-driven micellar transition pathway of monorhamnolipids. J. Colloid. Interf. Sci..

[39] Hoogerbrugge P.J., Koelman J.M.V.A. (1992). Simulating microscopic hydrodynamic phenomena with dissipative particle dynamics. Europhys. Lett..

[40] Koelman J.M.V.A., Hoogerbrugge P.J. (1993). Dynamic simulations of hard-sphere suspensions under steady shear. Europhys. Lett..

[41] Español P., Warren P. (1995). Statistical mechanics of dissipative particle dynamics. Europhys. Lett..

[42] Groot R.D., Warren P.B. (1997). Dissipative particle dynamics: Bridging the gap between atomistic and mesoscopic simulation. J. Chem. Phys..

[43] Pagonabarraga I., Frenkel D. (2001). Dissipative particle dynamics for interacting systems. J. Chem. Phys..

[44] Trofimov S.Y., Nies E.L.F., Michels M.A.J. (2002). Thermodynamic consistency in dissipative particle dynamics simulations of strongly nonideal liquids and liquid mixtures. J. Chem. Phys..

[45] Groot R.D., Madden T.J. (1998). Dynamic simulation of diblock copolymer microphase separation. J. Chem. Phys..

[46] Maiti A., McGrother S. (2004). Bead–bead interaction parameters in dissipative particle dynamics: Relation to bead-size, solubility parameter, and surface tension. J. Chem. Phys..

[47] Jiménez-Ángeles F., Kwon H.-K., Sadman K., Wu T., Shull K.R., Olvera de la Cruz M. (2019). Self-Assembly of charge-containing copolymers at the liquid–liquid interface. ACS Cent. Sci..

[48] Oh T., Nagao M., Hoshino Y., Miura Y. (2018). Self-Assembly of a double hydrophilic block glycopolymer and the investigation of its mechanism. Langmuir.

[49] Schmidt B.V.K.J. (2018). Double hydrophilic block copolymer self-assembly in aqueous solution. Macromol. Chem. Phys..

[50] Prego C., Torres D., Fernandez-Megia E., Novoa-Carballal R., Quiñoá E., Alonso M.J. (2006). Chitosan–PEG nanocapsules as new carriers for oral peptide delivery: Effect of chitosan pegylation degree. J. Control. Release.

[51] Letchford K., Burt H. (2007). A review of the formation and classification of amphiphilic block copolymer nanoparticulate structures: Micelles, nanospheres, nanocapsules and polymersomes. Eur. J. Pharm. Biopharm..

[52] Li C., Tho C.C., Galaktionova D., Chen X., Kral P., Mirsaidov U. (2019). Dynamics of amphiphilic block copolymers in an aqueous solution: Direct imaging of micelle formation and nanoparticle encapsulation. Nanoscale.

[53] Zhang C.Y., Yang Y.Q., Huang T.X., Zhao B., Guo X.D., Wang J.F., Zhang L.J. (2012). Self-assembled pH-responsive MPEG-*b*-(PLA-*co*-PAE) block copolymer micelles for anticancer drug delivery. Biomaterials.

[54] Breiner U., Krappe U., Jakob T., Abetz V., Stadler R. (1998). Spheres on spheres—A novel spherical multiphase morphology in polystyrene-block-polybutadiene-block-poly(methyl methacrylate) triblock copolymers. Polym. Bull..

[55] Geng J., Liu B., Xu L., Hu F.-N., Zhu J.-J. (2007). Facile route to Zn-based II−VI semiconductor spheres, hollow spheres, and core/shell nanocrystals and their optical properties. Langmuir.

