# Supplementary material for: Mesoscale Simulations of pH-Responsive Amphiphilic Polymeric Micelles for Oral Drug Delivery
Source: Pharmaceutics. 2019 Nov 20;11(12):620. doi: 10.3390/pharmaceutics11120620 (PMC6956019; doi:10.3390/pharmaceutics11120620)
Supplement: Supplementary file 1 [file pharmaceutics-11-00620-s001.pdf]

pharmaceutics

MDPI

Supplementary Materials: Mesoscale Simulations of pH-Responsive Amphiphilic Polymeric Micelles for Oral Drug Delivery

Zhimin Wu, Manzhen Duan, Di Xiong and Can Yang Zhang

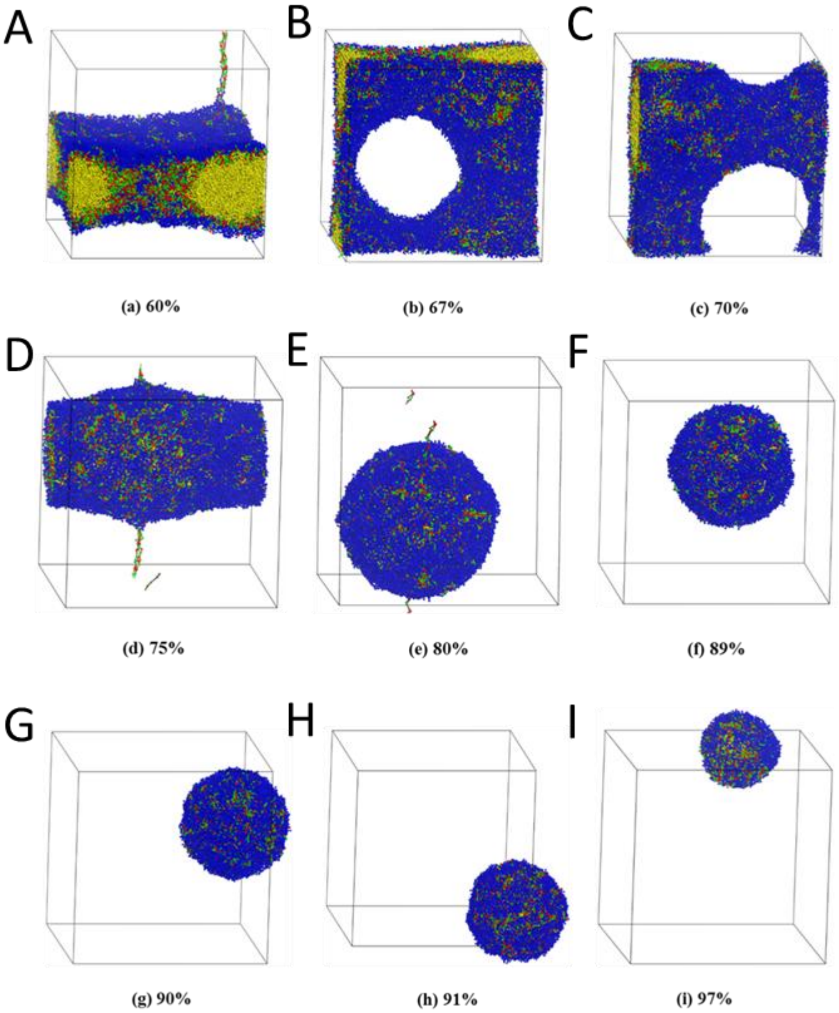

Figure S1. Morphologies of IBU-loaded polymeric NPs self-assembled from copolymer P(MMA_{30}-*co*-MAA_{33})-*b*-$PAEMA_{38}$ in aqueous with different mole fraction of water: fixed polymer : IBU = 8:2, adjust the molar ratio of water in the system to (**A**) 60%; (**B**) 67%; (**C**) 70%; (**D**) 75%; (**E**) 80%; (**F**) 89%; (**G**) 90%; (**H**) 91%; (**I**) 97%.

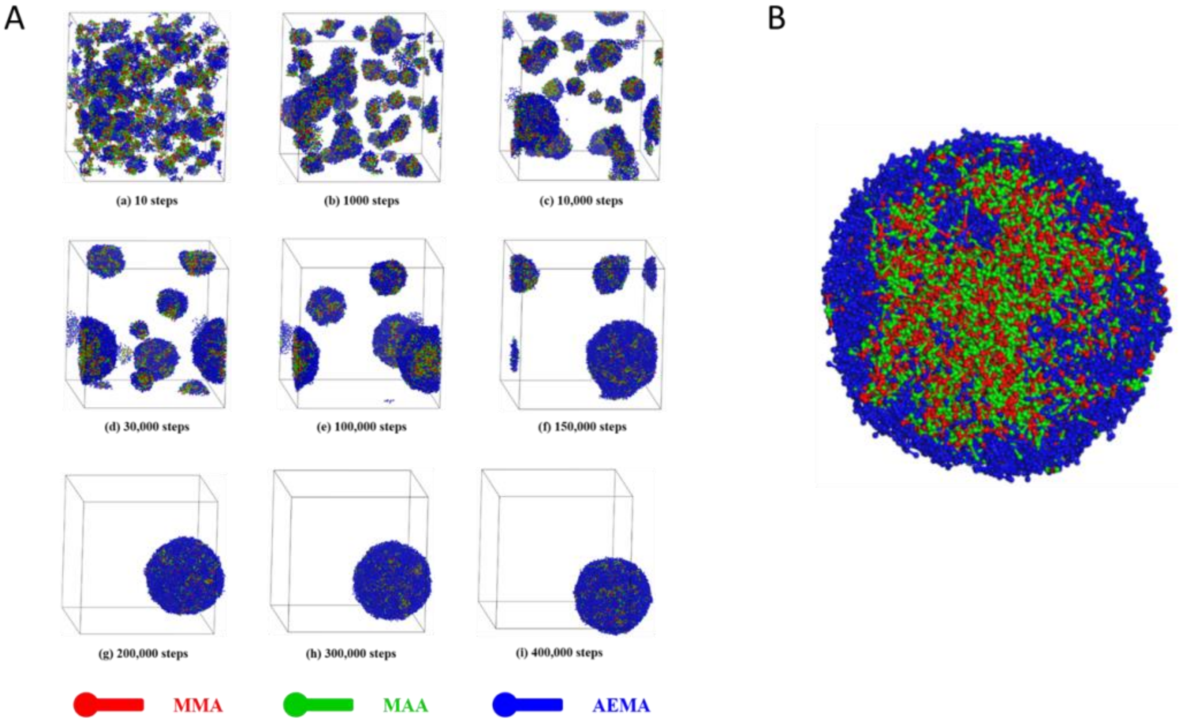

Figure S2. (**A**) Self-assembly process of copolymer P(MMA$_{30}$-*co*-MAA$_{33}$)-*b*-PAEMA$_{38}$ PMs in aqueous (**B**) Cross-section view of P(MMA$_{30}$-*co*-MAA$_{33}$)-*b*-PAEMA$_{38}$ PMs at 400,000 steps.

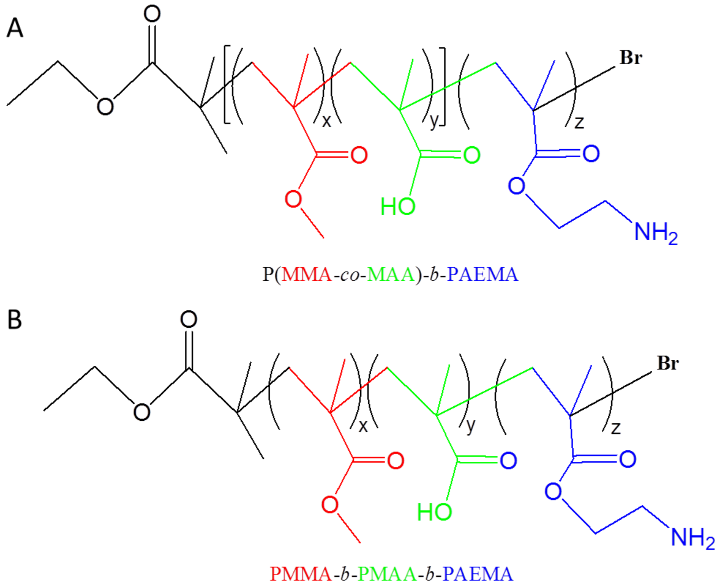

Figure S3. Chemical structures of random block copolymer P(MMA-*co*-MAA)-*b*-PAEMA (**A**) and precise line block copolymer PMMA-*b*-MAA-*b*-PAEMA (**B**).
